# Changes in Early Childhood Irritability and Risk-Taking on the Cambridge Gambling Task (CGT) at 11 Years

**DOI:** 10.1192/bjo.2024.249

**Published:** 2024-08-01

**Authors:** Ramya Srinivasan, Gemma Lewis, Francesca Solmi, Glyn Lewis

**Affiliations:** University College London, London, United Kingdom

## Abstract

**Aims:**

Irritability is common and easily identified in childhood. It is transdiagnostic and a common reason for referral to mental health services. Irritability which does not decrease during early childhood is associated with adolescent depression. We hypothesised that irritability would be associated with increased risk-taking overall but reduced risk-taking in response to loss.

**Methods:**

We used data from the Millennium Cohort Study, a population-based cohort of 18,552 children born in 2000–02. We examined whether irritability at 3, 5 and 7 years is associated with risk-taking on the CGT using multilevel mixed effect generalised linear models (MEGLMs). We also calculated the change in irritability between 3–7 years for each participant using multilevel mixed models. We then examined the association between this change measure and risk-taking on the CGT using MEGLMs. Analyses were adjusted for a broad range of confounders.

**Results:**

We found that children whose irritability did not decrease as would be expected from 3 to 7 years were more likely to stake a higher number of points per trial on the CGT at 11 years. This increase was most evident when the previous trial had been won. Irritability at 7 years was associated with staking a higher number of points per trial on the CGT (coefficient 0.52, 95%CI −0.04–1.08, p = 0.067) in fully adjusted model, whereas irritability at 3 and 5 years were not (3 years – coefficient 0.02, 95%CI -0.62–0.65, p = 0.961; 5 years – coefficient 0.14, 95%CI −0.45–0.73, p = 0.641). There was evidence of an interaction between irritability at seven years and whether the previous trial was won (p = 0.014). Childhood irritability which did not decrease between 3–7 years was associated with staking a higher number of points per trial on the CGT (coefficient 1.36, 95%CI 0.44–2.28, p = 0.004); there was evidence of an interaction between change in irritability and whether the previous trial was won (p = 0.056).

**Conclusion:**

This is the first longitudinal population-based study examining the relationship between changes in irritability during early childhood and risk-taking behaviour measured by the CGT. Our findings illustrate that irritability in children is characterised by an increase in risk-taking at age 11 years, reflecting differences in how children behave in relation to rewards and losses based on prior irritability. Further understanding of how the processes such as risk-taking which link childhood phenotypes such as irritability, relate to future mental health, may enable the development of new interventions focussing on reactions to rewards and losses.

